# A Simple Approach to Connecting Pt100 by Utilizing an Electroacoustic Resonance Tube

**DOI:** 10.3390/s23052775

**Published:** 2023-03-03

**Authors:** Mohamed Qawaqzeh, Farouq M. Al-Taweel, Kinga Stecuła, Katarzyna Markowska, Mohammad Al Khawaldah, Tariq M. Younes, Basem Alrifai, Oleksandr Miroshnyk, Taras Shchur

**Affiliations:** 1 Department of Electrical and Electronics Engineering, Al- Balqa Applied University, Al Salt 19117, Jordan; 2 Faculty of Engineering Technology, Department of Communication Engineering, Al- Balqa Applied University, Amman 11134, Jordan; 3 Department of Production Engineering, Faculty of Organization and Management, Silesian University of Technology, Akademicka 2a, 44-100 Gliwice, Poland; 4 Department of Transport Systems, Traffic Engineering and Logistic, Faculty of Transport and Aviation Engineering, Silesian University of Technology, 44-100 Gliwice, Poland; 5 Electrical Engineering Program, School of Engineering and Computing, American International University, Saad Al Abdullah-East of Naseem Block 3, Al Jahra 003200, Kuwait; 6 Department of Mehchatronics Engineering, Faculty of Engineering Technology, Al- Balqa Applied University, Al Salt 19117, Jordan; 7 Department Software Engineering, Al- Balqa Applied University, Al Salt 19117, Jordan; 8 Department of Electricity Supply and Energy Management, State Biotechnological University, 61052 Kharkiv, Ukraine; 9 GVA Lighting, Inc., Oakville, ON L6H 6X5, Canada

**Keywords:** acoustic resonance, temperature measurement, standing wave

## Abstract

Temperature transducers are frequently employed to keep track of process variables with different kinds of industrial controllers. One of the widely used temperature sensors is Pt100. A novel approach of utilizing an electroacoustic transducer in signal conditioning for Pt100 is proposed in this paper. A “signal conditioner” is a resonance tube filled with air, which is operated in a free resonance mode. The Pt100 wires are connected to one of the leads of the speaker in the resonance tube where the temperature changes, which is related to Pt100 resistance. The resistance affects the amplitude of the standing wave that is detected by an electrolyte microphone. An algorithm for measuring the amplitude of the speaker signal is described, as well as the building and functioning of the electroacoustic resonance tube signal conditioner. The microphone signal is acquired as a voltage using LabVIEW software. A virtual instrument (VI) developed under LabVIEW provides a measure of the voltage using standard VIs. The findings of the experiments reveal a link between the measured amplitude of the standing wave within the tube and the change in Pt100 resistance as the ambient temperature changes. Additionally, the suggested method may interface with any computer system when a sound card is added to it without the need for any extra measuring tools. The maximum nonlinearity error at full-scale deflection (FSD) is estimated at roughly 3.77%, and the experimental results and a regression model are used to assess the relative inaccuracy of the developed signal conditioner. When comparing the proposed approach with well-known approaches for Pt100 signal conditioning, the proposed one has several advantages such as its simplicity of connecting Pt100 to a personal computer directly via the sound card of any personal computer. In addition, there is no need for a reference resistance to perform a temperature measurement using such a signal conditioner.

## 1. Introduction

One of the most significant engineering tasks is still the measurement and control of temperature. This is because the efficiency and performance of technical mechanisms, machinery and systems are directly impacted by their temperature. This indicates why temperature measuring and control research and development is a valuable and ongoing activity [1].

Different types of transducers are used for temperature measurement and control. Each type of transducer has its own temperature-measuring parameters, as well as a somewhat complicated circuitry that is typically employed for signal conditioning and processing. There is still a need for a simple universal signal conditioner where temperature sensors can be interfaced directly to PC or microcomputers such as Raspberry PI or Arduino without the need for additional circuitry. This lowers the cost of the measurement and control circuit and equipment and unifies the grade and type of temperature transducers used [2,3].

This research paper presents an investigation into the possibility of using an electroacoustic transducer as a signal conditioner for one of the most common thermal resistors, namely Pt100.

Resistive elements are one of the most common types of sensors [4]. They are inexpensive and relatively easy to connect to normalizing circuits. Resistive elements can be made sensitive to temperature, deformation (under the action of force or bending) and the flow of light. Many complex physical processes can be measured using these elements, for example, the flow of liquids or masses (measuring the temperature difference of two calibrated resistors) [5].

A thermal resistive sensor is a parametric measuring sensor, the active resistance of which changes with temperature. Thermal resistive sensors can be formed from metal or semiconductor materials. The latter are called thermistors. The sensitive element of a metal thermoresistor is a thin copper or platinum wire [6]. The wire is wound bifilarly on a frame made of insulating and heat-resistant material. The sensitive element is placed in a metal protective sleeve (tube with a sealed end). Special terminals are available for connecting the thermoresistive sensors to the respective secondary device, which is called the signal conditioner. A simple signal conditioner can be made by utilizing a voltage divider, in which a thermoresistor is connected as one of the resistors of the driver, and the second resistive is a fixed resistor used as a reference. In the case of a voltage divider circuit, the temperature change is converted into resistance change, and this resistance change is produced as an output voltage of the voltage divider [7].

A more accurate approach to converting the temperature change of thermal resistance is applying a resistive bridge (such as a whetstone bridge). In this case, the thermal sensor is connected to one of the arms of the bridge and the measured voltage is a function of the temperature change [8]. The connection of thermal resistive sensors to the whetstone bridge can be classified according to the number of thermal sensors that are connected to each arm of the bridge, i.e., quarter, half and full. In some applications, it is recommended to connect the output voltage of the bride to a special type of amplifier circuit such as a differential amplifier or instrumentation amplifier for a more accurate solution. Nowadays, the signal conditioner of a thermal amplifier can be found as an integrated circuit, which is fabricated by several manufacturers [9]. 

Electroacoustic transducers are devices that convert electrical energy into acoustic energy (energy of elastic medium vibrations) and vice versa [10,11]. They identify transmitters and receivers based on the direction of conversion. Essentially, electroacoustic transducers perform two types of energy conversion [12,13]: electromechanical, in which a portion of the electrical energy supplied to the transducer is converted into the vibrational energy of a mechanical system, and mechanical-acoustic, in which a sound field is created in the medium as a result of mechanical system vibrations [14,15]. Sound receivers based on a change in the electrical resistance of a sensitive element under the influence of sound pressure [16], such as carbon microphones or semiconductor receivers that use strain measurement or the dependence of the semiconductor resistance on mechanical stresses [17,18], belong to a special class of electroacoustic transducers. When a transducer serves as a transmitter, an electric voltage (*V*) and current (*I*) are applied as its input, which determines its vibrational velocity (*c*) and sound pressure (*P*) when it is used as a receiver. However, pressure *P* or vibrational velocity *c* develops, resulting in voltage *V* and current *I* on its output [19]. Previous articles [20,21] describe the features of the electro-acoustic transducer–receiver:

- its sensitivity in the no-load and internal resistance *Z_in_* modes;
- its frequency dependence types (wideband and resonant receivers).

The operation of the electroacoustic transducer–transmitter is characterized by sensitivity, which is defined as a ratio that is dependent on several factors, including:

- the distance between the transmitter and the receiver;
- the current and voltage applied to the speaker coil;
- an electric energy source’s load, represented by internal resistance;
- the acoustic load resistance *Z_L_*, which is equal to the radiation resistance *Zr* when an electric field meets a continuous medium.

The stated parameters are frequency dependent. Electroacoustic transducers are commonly employed to generate and acquire sound. In ultrasonic technology, sonar and acoustoelectric, they are regarded as the “core” for measuring and receiving elastic vibrations. The most common electroacoustic transducers are linear, i.e., they meet the criteria for undistorted signal transmission, they are reversible, i.e., they can function as both a radiator and a receiver, and they follow the reciprocity principle.

The use of electroacoustic transducers to signal condition the Pt100 thermoresistor is proposed in this paper. In this scenario, the electroacoustic transducers are part of an air-filled resonance tube. When an acoustic wave is formed within the tube, the resistance of the thermoresistor is measured using the principle of amplitude variation. The temperature is then converted from the recorded amplitude.

Using phenomena based on acoustic wave propagation in a medium, many physical characteristics such as pressure, density, temperature and displacement could be evaluated. Acoustic field parameters such as amplitude, frequency and/or phase of an acoustic signal propagated in the material could be used to measure these quantities [22,23]. Temperature, pressure, displacement and density are among these variables [23,24,25,26]. For example, an acoustic temperature detector based on establishing a standing wave in a resonance tube was used to analyze the temperature in a heater [24], and the displacement of a moving spindle was recorded in the resonance mode of resonance tube operation [10]. In addition, researchers created a method for measuring the density of gases and vapors based on acoustic resonance [11].

Acoustic detectors are classed as velocity or impedance detectors based on the measured variable [11,12]. Time-impulse detectors and phase-interfering detectors are two types of velocity detectors. The time-based detectors work by detecting the time interval of an acoustic wave that has been transmitted and propagated through the medium. This time interval is considered a function of the measured value. The length of a propagated acoustic wave in an acoustic resonance tube is measured by phase-interfering detectors. The resonance frequency generated within a resonance tube is measured using both free resonance and a forced mode of operation [26].

The final required signal is frequency, according to a survey of existing acoustic detectors proposed in several works. The authors of [27] built software to measure the frequency of the captured signal. The authors used CBuilder Environment to display the link between the generated acoustic peak and time in their study, although the LabVIEW environment might also be used to measure the amplitude, frequency and phase of an acquired signal [28,29]. This means that extra instruments, such as a “frequency-to-voltage converter”, must be employed to display the desired signal.

These electronic circuits or signal processors are commonly used to build frequencies-to-voltage (*F*-to-*V*) converters [13]. A combination of electrical components, such as operational amplifiers, is used in the first technique. The frequency is converted into voltage using transistors and timers.

Previously, researchers used a U-shape resonance tube to assess the density of various liquids [28]. It was found that an acoustic wave propagating through a medium can provide information on the density of liquid.

In [30], the author presented a new signal conditioning circuit that uses a resonance tube to acquire LVDT signals. The signal conditioner was successfully employed to measure the disablement of the LVDT core, according to the experimental results.

## 2. Materials and Methods

### 2.1. Theoretical Background

The equation for displacement oscillations ξ in a pipe with gas under the adiabatic law is given by [31]

(1) $$\frac{\partial^{2}\xi }{\partial {\;t}^{2}}=c^{2}\frac{\partial^{2}\xi }{\partial {\;x}^{2}},$$

where c—the velocity of sound; 

ξ—the displacement of the particles of this section along the axis of the tube during vibrations;

x—the coordinate of the particles located at some cross-section of the pipe.

Equation (1) is called the wave equation. A particular solution of Equation (1) is a wave traveling along the *x* axis

*ξ* = *A*·cos(*ωt* − *kx* + *φ*), (2)

where *A*—the amplitude of the wave;

*ω*—the angular frequency;

*k = ω/c*—the wave number;

*ϕ*—the initial phase.

**Figure 1:**
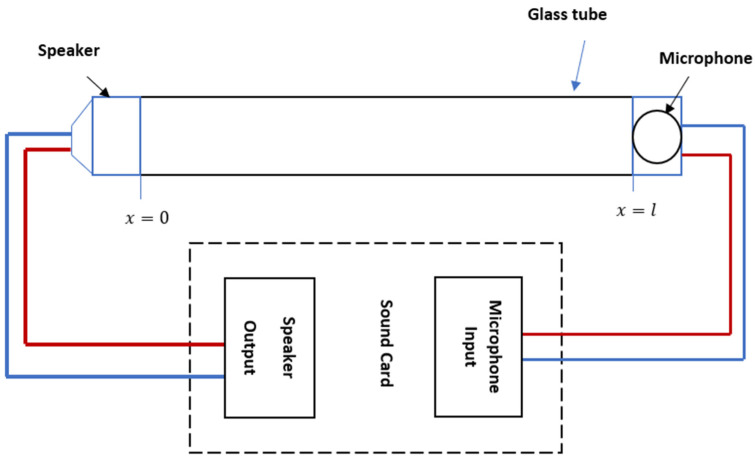
The shape of a resonance tube in which a standing wave is created.

Let a tube of length l filled with air be constructed in the form of a glass tube. The source of the acoustic wave is fixed at one of its ends, at point *x* = 0, and the microphone is fixed at point *x* = *l*, as shown in Figure 1.

In Figure 1, acoustic resonance occurs when a standing wave is generated in a tube filled with a medium such as air. The principle of acoustic resonance is often implemented by employing a speaker–microphone combination, with the two located at distance *L* from each other. An acoustic standing wave between the speaker and the microphone is generated using the sound card of the PC and transmitted within a tube. 

When resonance is created in the tube, two waves propagate in opposite directions at the same frequency. With continuous operation of the source, the wave traveling from it will be added to the reflected wave. For simplicity, we will assume that the reflected wave has almost the same amplitude as the incident one. By applying Equation (1), let us write the equations of the wave from the source *ξ*_1_ and the reflected wave *ξ*_2_ for a closed pipe [31]

(3) $$\xi_{1}=A\cos\left(\omega t-kx\right),$$

(4) $$\xi_{1}=A\cos\left(\omega t+kx+\pi \right).$$

As a result of the addition, the oscillation at point *x* will occur according to the following formula:

(5) $$\xi =\xi_{1}+\xi_{2}=2A\sin\left(kx\right)\sin\left(\omega t\right)=B\left(x\right)\sin\left(\omega t\right).\;$$

Thus, the oscillation amplitudes *B*(*x*) of different points of the resulting wave are different. The resulting wave is called a standing wave. Points whose oscillation amplitude is equal to zero are called nodes. We find the coordinates of the nodes from the equation

(6) B(x)=0 orsin(kx)=0. 

This equation has solutions

(7) $$x=\frac{\pi n}{k}=\frac{n\lambda }{2},\;n=0,1,\ldots \;$$

From Formula (7), it can be seen that the distance between neighboring nodes is equal to half the wavelength. Since there will be knots at the closed ends of the pipe, an integer number of half wavelengths *λ* fits on the length of the pipe *L*.

The wavelengths correspond to frequencies given by

(8) $$f_{n}=\frac{c}{\lambda_{n}}=\frac{c}{2L}n,\;n=1,2,\ldots \;$$

Harmonic vibrations with frequencies $f_{n}$ in Equation (8) are called natural or normal vibrations. They are also called harmonics.

Points that oscillate with maximum amplitude are called antinodes. We determine the antinode coordinates from the equation sin(kx)=±1:

(9) $$x=\left(n+\frac{1}{2}\right)\frac{\lambda }{2},\;\;\;n=1,2,\ldots \;$$

The distance between adjacent antinodes is also equal to half the wavelength.

**Figure 2:**
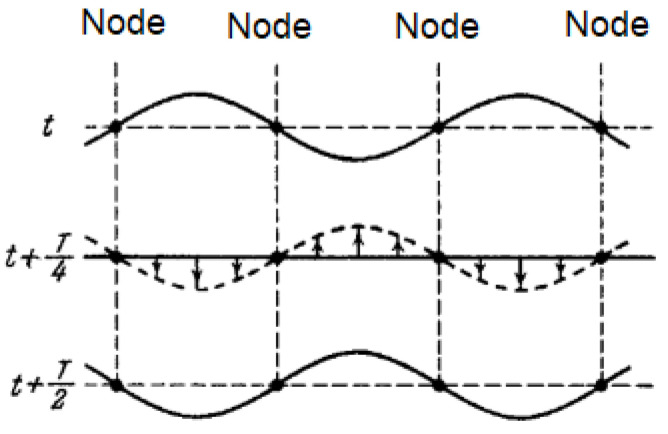
The presentation of modes.

The amplitude *B*(*x*) changes sign when passing through zero, which means that the points lying on opposite sides of the node oscillate in antiphase. All points enclosed between neighboring nodes oscillate in phase, as shown in Figure 2.

When we differentiate Equation (5) with respect to t, we find the expression for the particle velocity *∂t/∂ξ* of the gas

(10) $$\frac{\partial \xi }{\partial t}=2A\omega \sin\left(kx\right)\cos\left(\omega t\right).$$

When differentiating (5) with respect to *x* and applying formula $\frac{p^{\prime }-p}{p}=-\gamma \frac{\partial \xi }{\partial x}$ from [30], we obtain the sound pressure

(11) Δp=−2Aγpkcos(kx)sin(ωt),

where *γ*—the ratio of the heat capacity at constant pressure to the heat capacity at constant volume;

*p*—the pressure in initial conditions (if there are no oscillations of gas particles, then the pressure in the cross sections *x* and *x* + Δ*x* is the same and equals *p*).

The pressure difference obtained in Equation (11), can be measured by a microphone as a voltage output. 

### 2.2. Transducer Design

**Figure 3:**
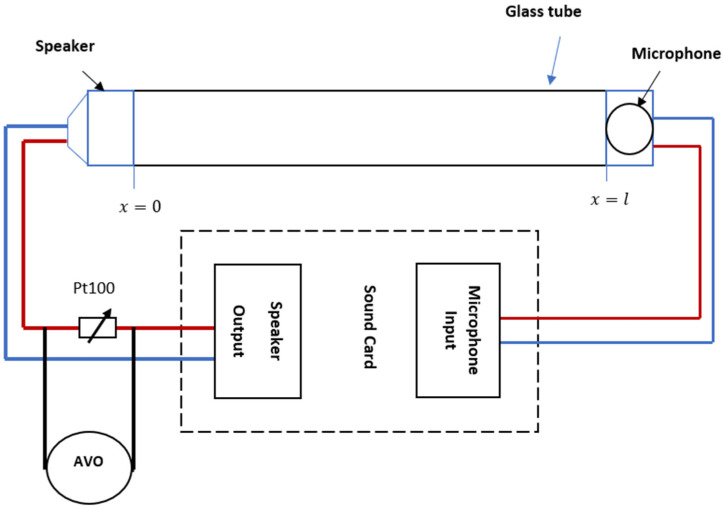
The connection lead wires with Pt100.

An electroacoustic resonance tube is a glass tube with an acoustic receiver attached to one end and an acoustic transmitter attached to the other end (Figure 3). 

The acoustic transmitter is a source of oscillation connected to a sound card’s speaker output or a PC or laptop’s speaker output. A microphone is connected to a sound card’s MIC input, and it is referred to as an acoustic oscillation receiver. Based on [10], there are two modes of resonance that can be formed within the resonance tube. The first mode is called free acoustic resonance. Without adjusting the resonance tube parameters, a standing wave is formed in this mode (frequency, amplitude, phase). The second mode is called a forced mode of resonance, and it is created by adjusting the tube’s parameters (specifically, the frequency value) in relation to the tube’s length and the type of gas it contains.

The operation of the electroacoustic resonance tube signal conditioner is shown below. When a standing wave is generated within a tube, the parameters of an electroacoustic resonance tube are extremely important. These are the parameters:

(1) Standing wave frequency;
(2) Standing wave amplitude;
(3) Standing wave phase;
(4) Voltage applied to the microphone or speaker;
(5) Current sent through the speaker or microphone.

In this work, the resonance tube will be operated as a signal conditioner for resistive-type sensors, namely Pt100. Figure 3 illustrates the speaker’s connection with Pt100.

The current flows via the resistance *R_t_*, and the coil of the speaker *X_L_* is given by the following formula

(12) $$I=\frac{V_{sp}}{X_{L}+R_{t}},$$

where *V_sp_*—the output voltage of the speaker;

*X_L_*—the inductive reactance of the coil;

*R_t_*—the temperature of Pt100 at temperature *t*.

Meanwhile, the impedance $X_{L}=\omega L=2\pi fL$, so Formula (12) can be written as

(13) $$I=\frac{V_{sp}}{2\pi fL+R_{t}},$$

where *L*—the inductance of the speaker circuit.

An electrodynamic speaker with an elastic membrane can be considered an inductive transducer, and the following expression is used to describe the relationship between the coil’s inductance and the produced pressure [15]

(14) $$L=\frac{\mu_{0}N^{2}A}{\delta_{0}},$$

where *μ*_0_—the relative magnetic permittivity of the air;

*N*—the number of turns of the coil;

*A*—the area of the membrane;

*δ*_0_—the value of the air gap between the membrane and the coil at temperature *t*_0_. 

By using Formula (4) in Formula (13), we can produce

(15) $$I=\frac{V_{sp}}{2\pi f\;\frac{\mu_{0}N^{2}A}{\delta_{0}}+R_{t}}.\;$$

Considering that $m=2\pi \;\frac{\mu_{0}N^{2}A}{\delta_{0}}$ = const, Equation (4) has the form

(16) $$I=\frac{V_{sp}}{f\cdot K+R_{t}}.$$

By rearranging Equation (5) according to *R_t_*, we can produce

(17) $$R_{t}=\frac{V_{sp}}{I}-f\cdot m.$$

Assuming that $a=\frac{1}{I}$, b=−f·m, Formula (17) can be written as

(18) $$R_{t}=a\cdot V_{sp}+b.$$

The value of *R_t_* can be calculated using Equation (8) based on the measured *V_sp_*, which is a sound oscillation acquired by the microphone (*V_MIC_*).

Based on the microphone’s physical attributes and design, manufacturers set the maximum decibel level. The maximum dB level is defined as the level at which the diaphragm approaches the backplate or at which the total harmonic distortion (THD), which is commonly 3 percent THD, reaches the prescribed level. The highest decibel level that a microphone may produce in each application relies on the voltage used and the sensitivity of that specific microphone. Calculating the pressure in pascals that the microphone can withstand is necessary before determining the maximum output for a microphone utilizing a given preamplifier with an accompanying peak voltage. Using the formula below, you can determine the pressure [32,33,34,35,36]:

(19) $$\Delta p=\frac{V_{MIC}}{S},\;$$

where Δp—equals the pressure of the wave at the antinodes, in (*P_a_*);

*V_MIC_*—the voltage produced by the microphone output (mV), which is approximately equal to half the amplitude of the voltage produced by the speaker when the speaker is adjusted to generate a voltage between –1 and +1 V;

*S*—the sensitivity of the microphone, mV/Pa. 

From Equation (19), we can obtain the voltage produced by the microphone when the sensitivity of the microphone is known, so Equation (11) can be written as

(20) $$V_{MIC}=\Delta p\cdot Sensitivty=-2\cdot A\cdot \gamma \cdot p\cdot k\cdot cos\left(x\right)\sin\left(\omega t\right).$$

So, the microphone measures the double amplitude, which is generated by the speaker in the resonance mode of operation when a standing wave is generated within a tube, and Formula (8) can be written as

(21) $$R_{t}=\frac{1}{2}aV_{MIC}+b.$$

Formula (9) supports the determination of the resistance of Pt100, which is defined by the following formula

(22) $$R_{t}=R_{0}\left(1+\alpha T\right).$$

So, to find the temperature from Equation (10), we can use the following

(23) $$T=\frac{\frac{R_{t}}{R_{0}}-1}{\alpha }.$$

Considering that $R_{t}=\frac{1}{2}aV_{MIC}+b$, as obtained from Equation (19), we can find the final relationship for the temperature change with the voltage output of the electroacoustic signal conditioner, i.e.,

(24) $$T=\frac{\frac{\frac{1}{2}aV_{MIC}\;\;+b}{R_{0}}-1}{\alpha }.$$

Equation (24) presents the relationship, which can be considered a mathematical model of the signal conditioner output voltage. It translates the change of temperature of thermal resistor Pt100 into voltage without the need for traditional circuitry such as a voltage divider *V_divider_* or whetstone bridge. A possible source of error in the proposed schemes is presented by the surrounding acoustic features of the environment; the lead wires effect of Pt100 needs to be considered and compensated for more accurate modeling.

### 2.3. Software Design

The LabVIEW programming environment was chosen for software development since it met all the requirements for sound measuring characteristics such as amplitude, frequency and phase [16].

**Figure 4:**
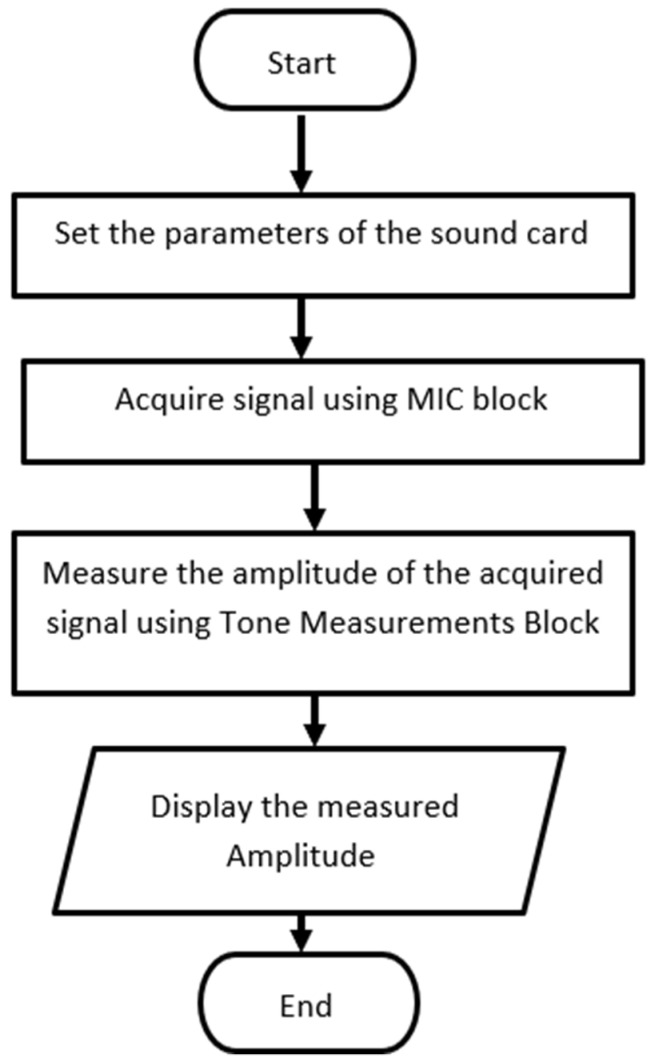
The algorithm for measuring the amplitude of the sound card.

Figure 4 shows the algorithm for measuring the amplitude of the speaker signal.

**Figure 5:**
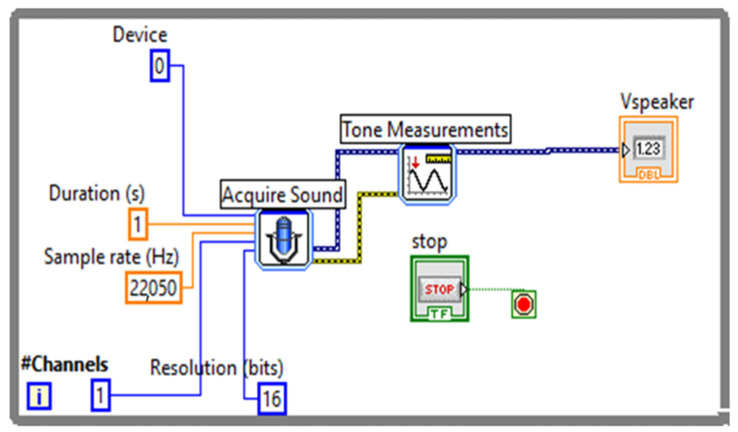
Simple virtual instrument (VI) to acquire the microphone signal.

Figure 5 depicts the development of a simple virtual instrument (VI) in LabVIEW to acquire the microphone signal, followed by the measurement of the microphone’s amplitude using the Tone Measurements block.

The advantage of using the resonance mode is that the transducer parameters, such as tube length and medium within the tube, are included in the measurement, eliminating the requirement for transducer calibration. The proposed approach of signal conditioning can find applications in gas chromatography, where acoustic detectors are used [27,30], to ensure the affecting temperature can be measured and controlled at the same time as the detection process. In addition, a new detector for gas chromatography can be designed using an acoustic detector and katharometer, where the chromatogram can be presented as a function of sound pressure and the change of katharometer resistance. 

## 3. Experimental Setup

**Figure 6:**
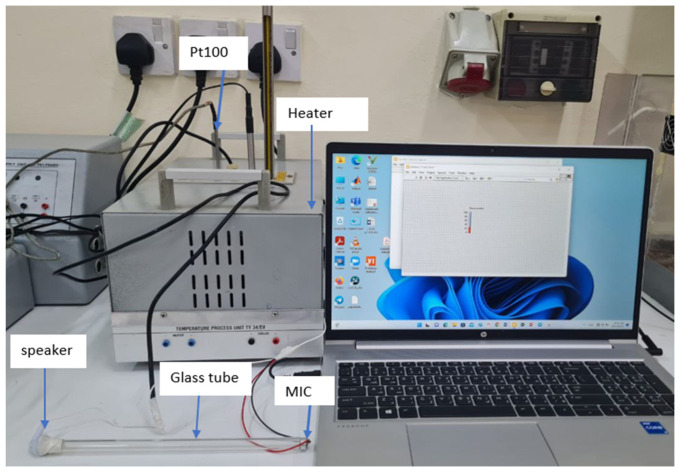
The experimental setup.

The experimental setup is shown in Figure 6. It consists of a heater system, along with an electroacoustic signal conditioner built from a glass tub, speaker and microphone.

## 4. Experimental Results and Data Analyses

Table 1 shows the relationship between the temperature, the measured resistance of Pt100 and the voltage of the speaker when the proposed approach is utilized, as well as the relationship between the temperature and the voltage when a voltage divider *V_divider_* is used. 

The results obtained for the electroacoustic signal conditioner are acceptable when compared with the results obtained for the voltage divider. The main advantage of using the electroacoustic signal conditioner is that it allows us to obtain the results directly on the PC, without the need for additional signal processing elements such as DAQ devices.

To test the experimental reproducibility and justification for the proposed approach, we compare the results obtained for the proposed signal conditioner with those in [35], where a traditional approach was used. The main comparison points are listed in Table 2.

In Table 2, we can see that the proposed signal conditioner has several advantages compared to the method proposed in work [35], such as the simplicity of connecting to any PC sound card and the lack of a need for additional processing blocks.

**Figure 7:**
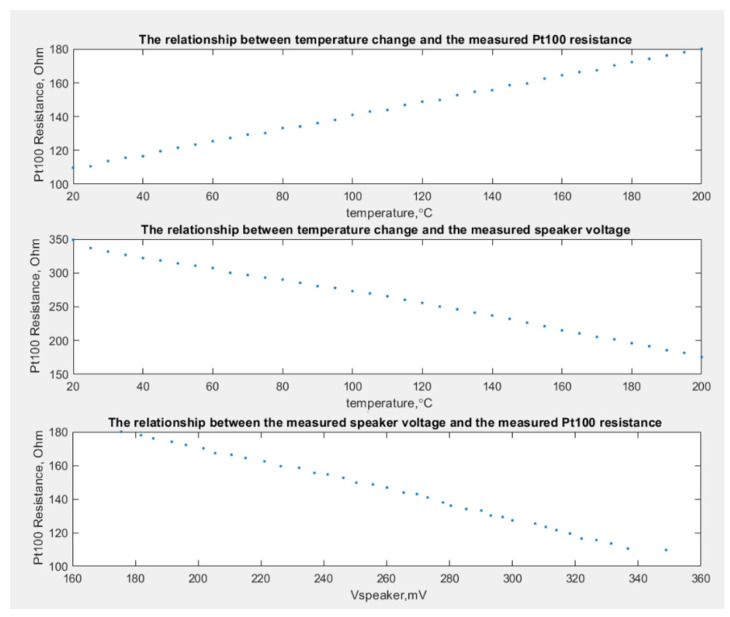
Plots between the temperature change, the measured Pt100 resistance and the measured speaker voltage.

Figure 7 shows plots between the temperature change, the measured Pt100 resistance and the measured speaker voltage.

The findings of the experiments reveal a relationship between the measured amplitude of the standing wave within the tube and the change in Pt100 resistance as the ambient temperature changes.

The cftool is used in MATLAB to calculate the least square regression line, which has a form similar to Equation (21)

*R_t_*(*V_speaker_*) = *p*_1_ꞏ*V_speaker_* + *p*_2_. (25)

The coefficients (with 95% confidence bounds) were found to be *p*_1_ = −0.4298 and *p*_2_ = 257.1. 

**Figure 8:**
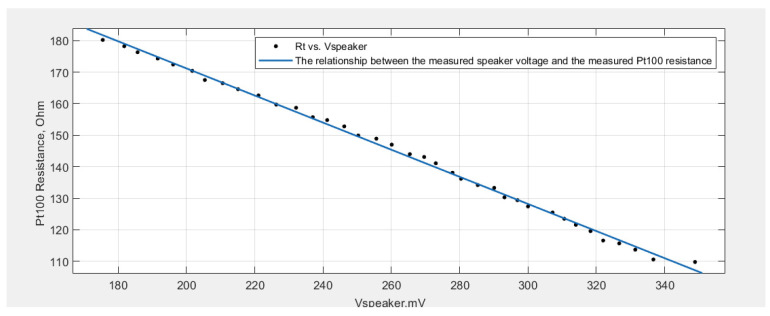
The curve fitting using cftool.

Figure 8 plots the curve fitting (calibration curve).

Regarding the calibration curve obtained using curve fitting tools, the nonlinearity is around 3.77%, which is acceptable for such a type of measurement. In order to achieve lower nonlinearity and high resonance frequency, air may be substituted with a gas that has a smaller molecular weight (M) and a higher specific heat ratio.

## 5. Conclusions

Previous studies showed how a resonance tube can be modified to measure a variety of physical and physical-chemical quantities. This paper demonstrates how to use a resonance tube as a signal conditioner to measure the resistance and temperature of Pt100. It was demonstrated experimentally that the measured resistance of Pt100 and the amplitude of the generated standing wave within a resonance tube have a linear relationship. The relationship between the temperature change and the measured voltage has a linear characteristic, where the nonlinearity error is about 2.8%. In addition, the proposed approach does not need any added measurement steps to perform the interfacing with a PC (or any computer system) via the sound card. When compared to other methods for Pt100 signal conditioning, the proposed methodology has several advantages, such as the ability to connect Pt100 directly to any personal computer’s sound card. Moreover, utilizing such a signal conditioner for temperature monitoring eliminates the requirement for a reference resistance.

## Figures and Tables

**Table 1:** Experimental data.

Surrounding Temperature, °C	Experimental Results		
	*R_t_*_,_ Ohm	*V_divider_*, V	*V_sp_*, mV
**20**	109.8	2.59	348.9
**25**	110.6	2.72	336.7
**30**	113.7	2.64	331.4
**35**	115.7	2.76	326.7
**40**	116.6	2.68	322.0
**45**	119.6	2.80	318.3
**50**	121.6	2.72	314.0
**55**	123.5	2.84	310.6
**60**	125.5	2.76	307.2
**65**	127.4	2.88	300.0
**70**	129.4	2.80	296.9
**75**	130.3	2.92	293.1
**80**	133.3	2.84	290.1
**85**	134.2	2.86	285.3
**90**	136.2	2.87	280.4
**95**	138.1	2.99	277.9
**100**	141.1	3.01	273.0
**105**	143.1	3.03	269.6
**110**	144.0	3.04	265.4
**115**	147.0	3.06	260.1
**120**	148.9	3.08	255.6
**125**	149.9	2.99	250.3
**130**	152.8	3.11	246.2
**135**	154.8	3.12	241.2
**140**	155.7	3.14	237.0
**145**	158.7	3.05	232.1
**150**	159.7	3.17	226.3
**155**	162.6	3.08	221.1
**160**	164.6	3.20	215.1
**165**	166.5	3.21	210.6
**170**	167.5	3.12	205.4
**175**	170.4	3.14	201.7
**180**	172.4	3.15	196.1
**185**	174.3	3.26	191.6
**190**	176.3	3.18	185.7
**195**	178.2	3.29	181.8
**200**	180.2	3.30	175.5

**Table 2:** Comparison of the proposed signal conditioner with the traditional approach in [35].

Comparison Parameter	Electroacoustic Signal Conditioner	Traditional Approach
Output signal	Volts	Volts
Need for reference resistor(s)	No need	1 resistor for voltage divider circuit. 3 resistors for whetstone bridge circuit
V	2 V	5 V
Need for special computer interfacing block	Does not need additional blocks Standard PC sound card can be implemented	Needs interfacing units to complete connection with PC
Range of measured temperature	20–100 °C	0–850 °C
Sensitivity	−7.44 mV/°C	5.88 mV/°C

## Data Availability

The data presented in this study are available on request from the corresponding author.
